# Nanoscale Insights into the Mechanical Behavior of Interfacial Composite Structures between Calcium Silicate Hydrate/Calcium Hydroxide and Silica

**DOI:** 10.3390/nano13233059

**Published:** 2023-11-30

**Authors:** Jiuye Zhao, Yuanhang Zhang, Dapeng Xue, Chunyi Cui, Wenzheng Li, Fang Liu

**Affiliations:** 1 College of Transportation Engineering, Dalian Maritime University, Dalian 116026, China; 2 School of Civil Engineering, Dalian University of Technology, Dalian 116024, China; 3 Shaanxi Key Laboratory of Safety and Durability of Concrete Structures, Xijing University, Xi’an 710123, China

**Keywords:** nanomechanical behavior, molecular dynamics, interfacial structure, hydration products

## Abstract

The failure of the interfacial transition zone has been identified as the primary cause of damage and deterioration in cement-based materials. To further understand the interfacial failure mechanism, interfacial composite structures between the main hydration products of ordinary Portland cement (OPC), calcium silicate hydrate (CSH) and calcium hydroxide (Ca(OH)_2_), and silica (SiO_2_) were constructed while considering their anisotropy. Afterwards, uniaxial tensile tests were conducted using molecular dynamics (MD) simulations. Our results showed that the interfacial zones (IZs) of interfacial composite structures tended to have relatively lower densities than those of the bulk, and the anisotropy of the hydration products had almost no effect on the IZ being a low-density zone. Interfacial composite structures with different configurations exhibited diverse nanomechanical behaviors in terms of their ultimate strength, stress–strain relationship and fracture evaluation. A higher strain rate contributed to a higher ultimate strength and a more prolonged decline in the residual strength. In the interfacial composite structures, both CSH and Ca(OH)_2_ exhibited ruptures of the Ca-O bond as the primary atomic pair during the tensile process. The plastic damage characteristics of the interfacial composite structures during the tensile process were assessed by analyzing the normalized number of broken Ca-O bonds, which also aligned with the atomic chain break characteristics evident in the per-atom stress map.

## 1. Introduction

Cementitious materials, including concretes, mortars and stabilized soils, etc., play a pivotal role in construction endeavors due to their inherent properties [1,2,3,4,5]. Within these materials, the process of cement hydration results in the formation of a cohesive structure by binding together the dispersed aggregates. This phenomenon simultaneously leads to the creation of an interfacial transition zone (ITZ) between the hydration products and aggregates [6,7]. Critically, the failure of this ITZ has been identified as the primary cause of damage and deterioration in cementitious materials [8,9]. Consequently, there has been a concerted effort within the scientific community to thoroughly analyze the distinctive characteristics of the ITZ and investigate the underlying mechanisms of its damage progression. In the pursuit of understanding the interfacial properties of cementitious materials, numerous scholars have employed experimental testing methods, such as nanoindentation, to probe the ITZ in concrete [10,11,12]. While these techniques provide valuable insights into the properties of the ITZ in cementitious materials, they fall short in elucidating the intricate nanoscale processes and mechanisms governing interface degradation.

With the continuous advancement of computer simulation technology, a variety of microsimulation methods can effectively support the study of cementitious materials at the molecular scale, among which molecular dynamics (MD) has been widely adopted [13,14,15,16]. In the study of cementitious materials involving MD, calcium silicate hydrate (hereafter abbreviated as CSH), the main constituent of hydrated ordinary Portland cement (OPC), has attracted much attention. The strength, modulus, stress–strain relationship and failure mechanism of CSH under tensile conditions have been systematically investigated. It was found that factors such as the calcium–silicon ratio, water content and ion exchange can affect the nanomechanical behavior of CSH [17,18]. Furthermore, during the simulation process, CSH exhibits noticeable heterogeneous characteristics that exert a substantial influence on its properties at both the molecular and mesoscopic scales [19,20,21].

In more recent studies, MD has been explored for analyzing the interfacial interaction between cementitious materials and other materials at the nanoscale. A series of research studies focused on the interfacial interaction between CSH and reinforced materials, such as graphene oxide, carbon nanotubes, fibers and epoxy resin [22,23,24]. The bonding properties of the interface at the molecular level have been evaluated by tensile tests and pull-out tests [22,25,26]. The influence of factors, including moisture, defects and environmental impacts, on the bonding behavior of the interface was investigated [16,24,26]. Meanwhile, to unravel the nanomechanical behavior of the ITZ and understand the interaction mechanism, a few studies have attempted to analyze the interface between hydration products and aggregates. Kai et al. analyzed the diffusion behavior, bond strength and fracture process of the interface between CSH gel and silica, and the result showed that a higher water content in the CSH gel reduces the interfacial interactions [27]. Through uniaxial tensile tests in MD simulations, Wu et al. proved that increasing ITZ thickness negatively impacted the mechanical properties of a CSH-SiO_2_ system [28]. The influence of moisture on the interface of CSH-calcite/silica was investigated by Zhou et al., and the results indicated that increased moisture at the interface and a slower loading rate were associated with decreased adhesion strength [29]. As for the interfacial characteristics of a geopolymer binder to aggregate composites, Kai and Dai found that the interfacial fracture undergoes three stages, including crack propagation, chain bridging and breakage, during the loading process [30]. These studies provided insights into the understanding of nanoscale interactions between hydrated pastes and aggregates, but further investigations are still needed to address the following two unresolved issues. Firstly, the effect of the asymmetric structure of CSH on the interfacial structure needs to be urgently evaluated, as CSH exhibits significant heterogeneous nanomechanical behaviors. In addition, calcium hydroxide (Ca(OH)_2_), another major hydration product of OPC cements, is similarly distributed in large quantities on the aggregate surface and forms interfacial structures in cementitious materials. However, the interfacial bonding properties of CSH and calcium hydroxide have not been fully analyzed in a comparative manner.

In this study, the molecular models of CSH and Ca(OH)_2_ were respectively cleaved along crystallographic planes of (100), (010) and (001) to construct interfacial composite structures with SiO_2_ (the major mineral of aggregates/fillers in mortars and cemented soils) to consider the anisotropy of hydration products at the nanoscale. Additionally, uniaxial tensile tests were conducted using MD simulations. Based on results of the density profiles, the stress–strain relationships and fracture patterns, the anisotropic nanomechanical behaviors of the composite structures of SiO_2_-CSH-SiO_2_ and SiO_2_-Ca(OH)_2_-SiO_2_ were systematically compared. Additionally, a normalized number of broken bonds and per-atom stress mapping on the local scale were combined to study the fracture mechanism of interfacial composite structures with different configurations. The results of the current study can provide comprehensive insights into the nanoscale mechanical behavior of the ITZ.

## 2. Computational Method

### 2.1. Simulation Model

In terms of modelling, a supercell of monoclinic quartz (a = b = 4.9 Å, c = 5.4 Å, α = β = 90°, γ = 120°) was converted into an orthorhombic structure to form the SiO_2_ layer in the interfacial composite structure. The method suggested by Pellenq et al. was used to construct the C-S-H cell model [31]. The 11 Å tobermorite configuration, as proposed by Hamid, served as the foundation for constructing the CSH model after eliminating the initial water content. Subsequently, water adsorption was performed in the interlayer space by randomly removing SiO_2_ units through the application of the Grand Canonical Monte Carlo (GCMC) simulation technique. The *Q*_n_ distributions of the silicate chains in the CSH molecule used in this paper were *Q*_0_ = 9.4%, *Q*_1_ = 68.2% and *Q*_2_ = 22.4%, which are close to the distributions of the experimental samples (*Q*_0_ = 10%, *Q*_1_ = 67%, *Q*_2_ = 23%) [32]. Furthermore, the influence of the oxhydryl (-OH) group on the mechanical properties of CSH was not taken into account here due to its limited effect [33]. The saturated C-S-H structure exhibits the chemical formula of (CaO)_1.69_(SiO_2_)·1.81H_2_O, which closely resembles the composition (CaO)_1.7_(SiO_2_)·1.8H_2_O obtained through the small-angle neutron scattering (SANS) test [34]. The supercell of the CSH molecule was cleaved along the (100), (010) and (001) crystallographic planes, respectively, to form the CSH layers in composite models. After orthogonally transforming, the supercell of a monoclinic crystal system Ca(OH)_2_ (a = b = 3.56 Å, c = 4.88 Å, α = β = 90°, γ = 120°) was cleaved along the (100), (010) and (001) crystallographic planes, respectively, to form the Ca(OH)_2_ layers in composite structures.

**Figure 1:**
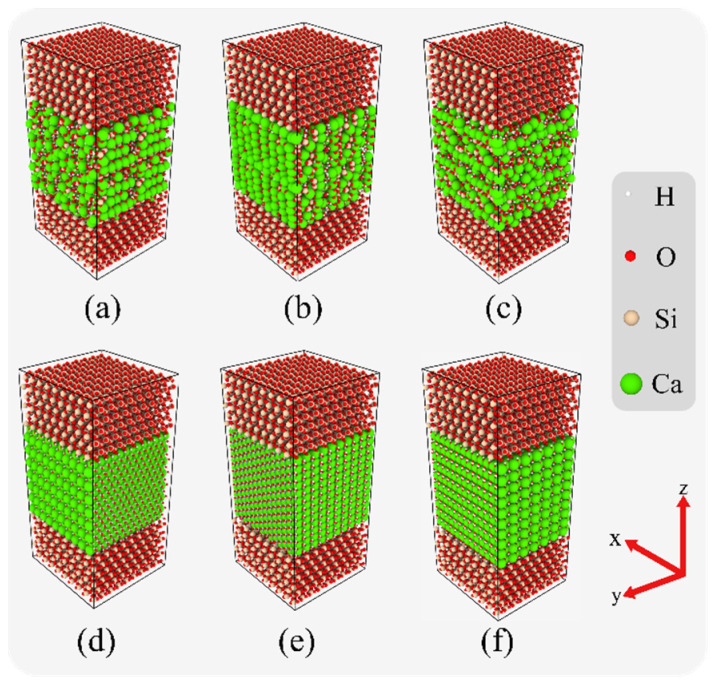
Perspective views for different configurations of the interfacial composite structures, (**a**) SiO_2_-CSH_(100)-SiO_2_, (**b**) SiO_2_-CSH_(010)-SiO_2_, (**c**) SiO_2_-CSH_(001)-SiO_2_, (**d**) SiO_2_-Ca(OH)_2__(100)-SiO_2_, (**e**) SiO_2_-Ca(OH)_2__(010)-SiO_2_, and (**f**) SiO_2_-Ca(OH)_2__(001)-SiO_2_.

In the assembled interfacial composite structures, the silica layer, the hydration product layer (CSH or Ca(OH)_2_) and the silica layer, in order from top to bottom, have a thickness ratio of 1:2:1. The dimensions of the interfacial composite structures were in the range of (44.7 ± 1.0) Å × (47.7 ± 1.0) Å × (99.2 ± 3.0) Å. In the subsequent discussion, the notations SiO_2_-CSH-SiO_2_ and SiO_2_-Ca(OH)_2_-SiO_2_ are employed to represent the interfacial composite structures consisting of CSH and Ca(OH)_2_, respectively. Furthermore, the notations SiO_2_-CSH_(hkl)-SiO_2_ and SiO_2_-Ca(OH)_2__(hkl)-SiO_2_ are utilized to denote specific configurations of the interfacial composite structure where the hydration product interface is situated on the (hkl) crystallographic plane. Perspective views for different configurations of the interfacial composite structures are shown in Figure 1.

### 2.2. Molecular Dynamics Modeling

The constructed interfacial composite structure was loaded into the Large-scale Atomic/Molecular Massively Parallel Simulator (LAMMPS), and the clay force field (CLAYFF) was utilized to conduct uniaxial tensile simulation tests. The CLAYFF has demonstrated its effectiveness in analyzing the structural properties of oxide and hydroxide materials and has been widely adopted for accurately capturing intricate interactions involving calcium, silicate, oxygen and hydrogen atoms within the calcium silicate hydrate (CSH) system [29].

For all simulations, periodic boundary conditions (PBCs) were implemented in all directions of the interfacial composite structures. The Ewald method was used to calculate the long-range Coulomb force, and the integration of the equations of motion was completed using the Verlet algorithm with a time step of 1.0 fs. Interfacial composite structures were subjected to geometrical optimization through energy minimization employing the conjugate gradient (CG) algorithm, with an energy convergence criterion of 10^−6^ kcal/mol and a force convergence criterion of 10^−6^ kcal/(mol-Å). The NVT ensemble was initially employed for a relaxation period of 150 ps at 298 K, followed by the utilization of the NPT ensemble for an additional 150 ps at 298 K. The complete relaxation process of the model required a total simulation time of 300 ps to establish system equilibrium at a temperature of 298 K. The Nosé–Hoover thermostat was used to control the temperature, which accelerated the interfacial structure to equilibrium. The constant strain method was employed to apply uniaxial tensile loads, whereby the dimensions of the model along the z direction underwent linear variation over time. The strain rates were set at 0.08 ps^−1^, 0.008 ps^−1^ and 0.0008 ps^−1^, respectively. During simulations, the NPT ensemble was used at a temperature of 298 K and the pressure was controlled at 101 kPa in the x and y directions to take the Poisson effect into account.

### 2.3. Data Analysis

In order to ascertain the atomic displacement within the interfacial composite structures under tension, the mean squared displacement (MSD) of specific types of atoms was calculated using the following equation [35]:

(1) $$MSD(t)=\langle |r(t)-r_{0}{|}^{2}\rangle =\frac{1}{N}\sum_{i=1}^{N}\langle |r_{i}(t)-r_{i}(0){|}^{2}$$

where *N* represents the atom number to be averaged. *r_i_*(*t*) and *r_i_*(0) represent the position of the *i*-th atom at time *t* and 0, respectively.

In order to compare the number of broken bonds for different atom pairs in the interfacial composite structure during the tensile process, the density characteristics of the interatomic distance distribution were analyzed by the radial distribution function (RDF), and then the parameters for the normalized number of broken bonds were determined. The RDF for atoms *j* around *i* was calculated according to [36] as follows:

(2) $$g{\left(r\right)}_{ij}=\frac{n{\left(r\right)}_{j}}{\rho_{j}V}≐\frac{n{\left(r\right)}_{j}}{4\pi \rho_{j}r^{2}\Delta r}$$

where *n*(*r*)*_j_* represents the average number of atoms *j* around *i* in a spherical region of the radius from *r* to *r* + Δ*r*, and *ρ_j_* represents the number density of atoms *j*. *r* is the distance between atoms *i* and *j*.

The fracture normalization parameter is calculated according to [37] as follows:

(3) $$r_{\mathrm{bond}}=\frac{N_{0}-N}{N_{0}}\times 100\%=\frac{\Delta N}{N_{0}}\times 100\%$$

where *N* and *N*_0_ represent the numbers of a specific bond at a particular state under a given strain and the initial strain 0, respectively.

## 3. Results

### 3.1. Density Profile

**Figure 2:**
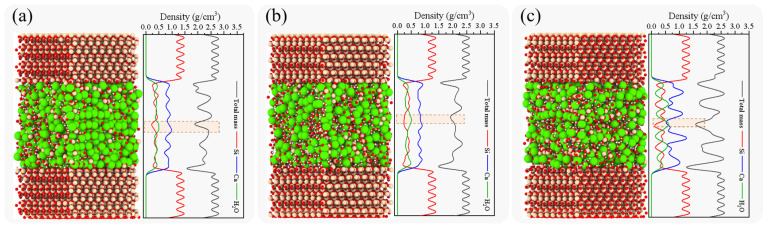
Density profiles of interfacial composite structures for SiO_2_-CSH-SiO_2_ in z direction, (**a**) SiO_2_-CSH_(100)-SiO_2_, (**b**) SiO_2_-CSH_(010)-SiO_2_, and (**c**) SiO_2_-CSH_(001)-SiO_2_. (red spheres represent the O atoms; yellow spheres represent the Si atoms; white spheres represent the H atoms; green spheres represent the Ca atoms).

Figure 2 shows the density profiles of the interfacial composite structures for SiO_2_-CSH-SiO_2_ in the z direction. It can be observed that although the CSH layers, cleaved along different crystallographic planes, showed obviously different density distributions, the interfacial zones (hereafter abbreviated as IZs) in all three composite structures tended to have relatively lower densities than those of the bulk. IZs with low-density phenomena have been mentioned in previous studies and can become potential fracture sites during loading [27,30]. It is shown here that the anisotropy of the CSH has almost no effect on the IZ being a low-density zone. Moreover, the interior of the CSH bulk displays a distinct layer-like structure with periodic density fluctuations. This layered structural characteristic of the CSH molecular model has also been observed in numerous previous studies, corroborating our experimental findings [31,33]. Notably, these studies have identified the regions where water molecules tend to aggregate during the stretching of CSH alone and its involvement in composite structures as potential fracture sites [18,38]. As depicted in the dashed regions of Figure 2a–c, the areas with elevated water densities tended to correspond to lower overall densities. Meanwhile, SiO_2_-CSH_(001)-SiO_2_ exhibited more concentrated regions of a higher water density than the other two configurations.

**Figure 3:**
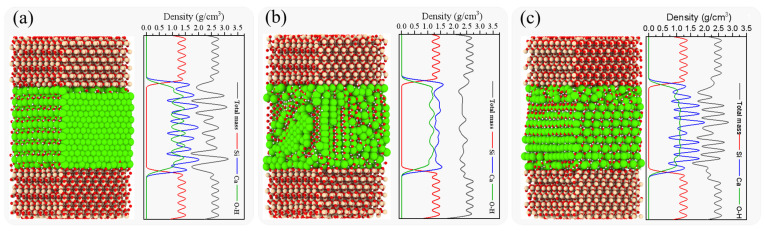
Density profiles of interfacial composite structures for SiO_2_-Ca(OH)_2_-SiO_2_ in z direction, (**a**) SiO_2_-Ca(OH)_2__(100)-SiO_2_, (**b**) SiO_2_-Ca(OH)_2__(010)-SiO_2_, and (**c**) SiO_2_-Ca(OH)_2__(001)-SiO_2_. (red spheres represent the O atoms; yellow spheres represent the Si atoms; white spheres represent the H atoms; green spheres represent the Ca atoms).

The density profiles of the interfacial composite structures for SiO_2_-Ca(OH)_2_-SiO_2_ in the z direction are displayed in Figure 3. Similar to the SiO_2_-CSH-SiO_2_ structure, the SiO_2_-Ca(OH)_2_-SiO_2_ composite structure usually exhibited relatively low densities in the interface region. Moreover, the density distributions of Ca^2+^ and hydroxyl groups (OH^−^) in the different configurations for both the interface and the Ca(OH)_2_ bulk differed significantly. This implies that while the symmetry of Ca(OH)_2_ can be reflected in a single configuration due to its crystalline nature, its anisotropic micromechanical behavior is likely to exist in composite structures with different configurations.

The low density observed in the IZ for all configurations can be attributed to the construction method of the interfacial composite structure. Specifically, during the construction process of the composite structure, there exists an initial thickness between the hydration product layer and the SiO_2_ layer, as detailed in reference [28], with the chosen initial thickness set to 3 Å in this study. The fracture surfaces of the simulated composite structures using this construction method closely resemble the observed phenomenon in macroscopic experiments on cemented soils, in which hydration products were observed to remain on the quartz surface [39]. Meanwhile, this initial thickness contributes to the lower density observed in the IZ region during the single-atom density calculation process, a characteristic that has been consistently reported in previous studies [27,28,30].

### 3.2. Nanomechanical Properties of Different Configurations

**Figure 4:**
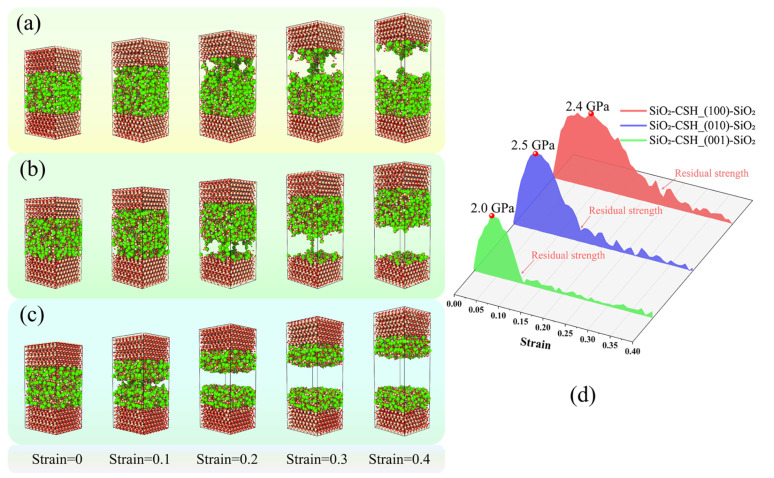
Fracture evolution for different configurations of SiO_2_-CSH-SiO_2_ at 0.008 ps^−1^, (**a**) perspective views of the fracture evolution of SiO_2_-CSH_(100)-SiO_2_, (**b**) perspective views of the fracture evolution of SiO_2_-CSH_(010)-SiO_2_, (**c**) perspective views of the fracture evolution of SiO_2_-CSH_(001)-SiO_2_, and (**d**) stress–strain curves. (red spheres represent the O atoms; yellow spheres represent the Si atoms; white spheres represent the H atoms; green spheres represent the Ca atoms).

In order to analyze the influence of the anisotropic molecular structure of the hydration products on the nanomechanical properties of the interfacial composite structures, the fracture process and the stress–strain relationship of different configurations are compared at the same strain rate. Considering the potential impacts of relatively high and low strain rates on fracture development and stress–strain curves, a moderate strain rate of 0.008 ps^−1^ was utilized for the analyses presented in this section. Figure 4 shows the fracture evolution of the structure of SiO_2_-CSH-SiO_2_ at 0.008 ps^−1^.

By integrating perspectives on fracture evolution and the stress–strain relationship during the tensile process, clear crack propagations in the descending section were evident following the attainment of ultimate stress; furthermore, the residual strength resulting from atomic chain bridging rapidly diminished and approached zero subsequent to the rupture of all atomic chains. The configuration influenced both the fracture site and characteristics of the interfacial composite structures. In terms of the fracture site, the SiO_2_-CSH_(100)-SiO_2_ and SiO_2_-CSH_(010)-SiO_2_ configurations (see Figure 4a,b) fractured within the interfacial zones with relatively low densities, whereas the SiO_2_-CSH_(001)-SiO_2_ configuration (see Figure 4c) fractured within the bulk of the CSH. With regard to their fracture characteristics, all three configurations exhibited significant differences in atomic bridging chains during the tensile process and the flatness of their respective fracture surfaces. The presence of substantial and uninterrupted atomic chain bridging led to a non-planar fracture surface rather than a flat plane, such as SiO_2_-CSH_(100)-SiO_2_. In the case of SiO_2_-CSH_(001)-SiO_2_, the fracture predominantly transpired within the water molecule layer of the CSH bulk, which can be attributed to the relatively weaker atomic chain bridging present between the water molecules. Consequently, the resulting fracture surface displayed a comparatively more orderly plane.

It is worth noting that, drawing on the findings from a prior investigation into the impact of various initial thicknesses (as reported in reference [28]) and taking into account the observed occurrence of surface-bound hydration product remnants on silica particles (as documented in reference [39]), the initial thickness between the hydration product layer and the SiO_2_ layer was controlled at 3 Å during the modelling process in this study. Therefore, even if a fracture occurs at the interface, a portion of the CSH atoms remain tightly adsorbed by the SiO_2_ following complete fracturing. This phenomenon has also been reported in the investigation of other interface models [27,28]. In terms of the stress–strain relationship (Figure 4d), the configuration had a substantial impact on both the ultimate stress and the decay process of the residual strength. In the SiO_2_-CSH_(100)-SiO_2_ structure, a notable number of atomic chains remained within the structure, continuing to serve as bridging agents even after the occurrence of noticeable cracks. As a result, the decay process of its residual strength can be characterized by a markedly sluggish rate. In SiO_2_-CSH_(010)-SiO_2_, only a limited quantity of atomic chains persisted after the evident damage of the composite structure. Therefore, the decay process of its residual strength occurred relatively swiftly. As for the atomic chains within the structure of SiO_2_-CSH_(001)-SiO_2_, their bridging effect was characterized as weak, owing to the interaction between water molecules. Consequently, the residual strength decay process exhibited the most rapid rate among the three configurations. In the discussion section, the bridging effects of various configurations of interfacial composite structures are further compared and discussed.

**Figure 5:**
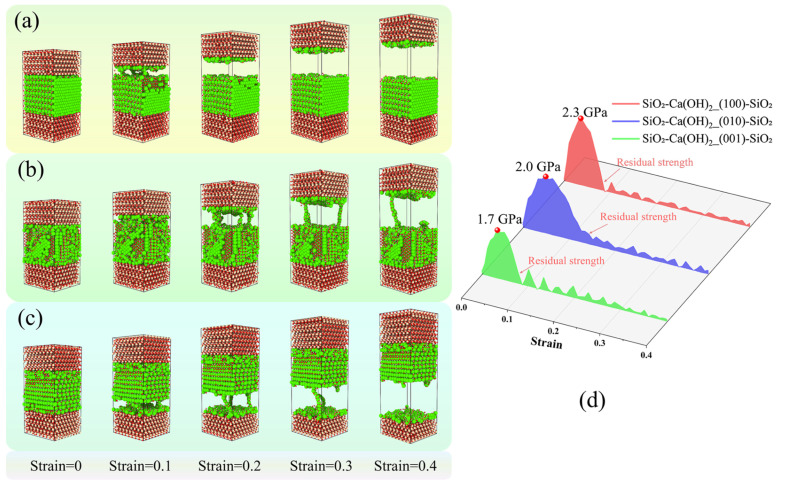
Fracture evolution for different configurations of SiO_2_-Ca(OH)_2_-SiO_2_ at 0.008 ps^−1^, (**a**) perspective views of the fracture evolution of SiO_2_-Ca(OH)_2__(100)-SiO_2_, (**b**) perspective views of the fracture evolution of SiO_2_-Ca(OH)_2__(010)-SiO_2_, (**c**) perspective views of the fracture evolution of SiO_2_-Ca(OH)_2__(001)-SiO_2_, and (**d**) stress–strain curves. (red spheres represent the O atoms; yellow spheres represent the Si atoms; white spheres represent the H atoms; green spheres represent the Ca atoms).

The fracture evolution for SiO_2_-Ca(OH)_2_-SiO_2_ at a strain rate of 0.008 ps^−1^ is shown in Figure 5. Based on perspective views of the fracture evolution (Figure 5a–c), in all configurations of SiO_2_-Ca(OH)_2_-SiO_2_, the fracture occurred in the region of the interfacial zone (IZ), which was characterized by a relatively low density. Notably, the fracture surfaces appeared relatively smooth and tidy. The observed phenomenon can be attributed to the crystal structure of calcium hydroxide, which consists of alternating calcium ions and hydroxyl groups. Localized bond breakage within this structure tended to lead to a concentration of stress in the surrounding region, ultimately resulting in the fracturing of other bonds within the same layer. With regard to the stress–strain behavior (Figure 5d), Ca(OH)_2_ also demonstrated distinct anisotropic micromechanical characteristics. Specifically, the SiO_2_-Ca(OH)_2__(010)-SiO_2_ configuration exhibited a relatively slower rate of residual strength degradation compared to the other two configurations. This observation can be attributed to the presence of a larger number of atomic chains facilitating bridging effects within this specific configuration.

**Figure 6:**
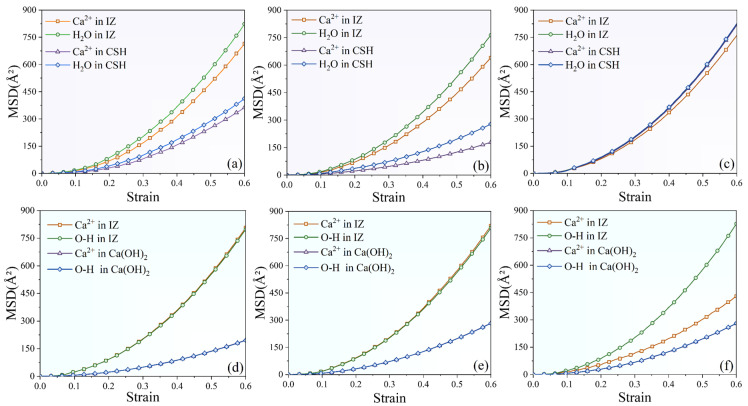
Mean squared displacement for different configurations of the interfacial composite structures, (**a**) SiO_2_-CSH_(100)-SiO_2_, (**b**) SiO_2_-CSH_(010)-SiO_2_, (**c**) SiO_2_-CSH_(001)-SiO_2_, (**d**) SiO_2_-Ca(OH)_2__(100)-SiO_2_, (**e**) SiO_2_-Ca(OH)_2__(010)-SiO_2_, and (**f**) SiO_2_-Ca(OH)_2__(001)-SiO_2_.

To identify the characteristics of atomic displacements in the IZ during the tensile process, MSD values for different configurations are compared in Figure 6. For both CSH and Ca(OH)_2_, the MSD values of the H_2_O molecular/OH^−^ group and Ca^2+^ ions in the IZ and overall exponentially increased with increasing strain, which indicates the state of the movement of the atoms during the interfacial composite structures’ tensioning. As for the configuration SiO_2_-CSH_(100)-SiO_2_ and SiO_2_-CSH_(010)-SiO_2_ which fractured at the interface (see Figure 6a,b), the MSD of the H_2_O molecules and Ca^2+^ ions in the IZ were significantly higher than those of the overall CSH. As for SiO_2_-CSH_(001)-SiO_2_ (fractured in the bulk of CSH) in Figure 6c, the MSD of the H_2_O molecules and Ca^2+^ ions in the IZ were almost equal to those of the overall CSH. With regard to all three configurations of Ca(OH)_2_ from Figure 6d–f, the OH^−^ groups and Ca^2+^ ions in the IZ showed larger MSD values compared to the total. To summarize, in scenarios in which the fracture took place in the interfacial region (excluding the SiO_2_-CSH_(001)-SiO_2_ configuration), the diffusion rates of the water molecules/OH^−^ groups and Ca^2+^ ions were notably higher in the interfacial region compared to the average rates observed in the entire layer of the hydration product. Conversely, when the fracture occurred within the hydration product (as in the SiO_2_-CSH_(001)-SiO_2_ configuration), the average diffusion rates of water molecules/OH^−^ groups and Ca^2+^ ions in the interfacial region and the overall layer of the hydration product were found to be essentially the same.

### 3.3. Influence of Strain Rate

**Figure 7:**
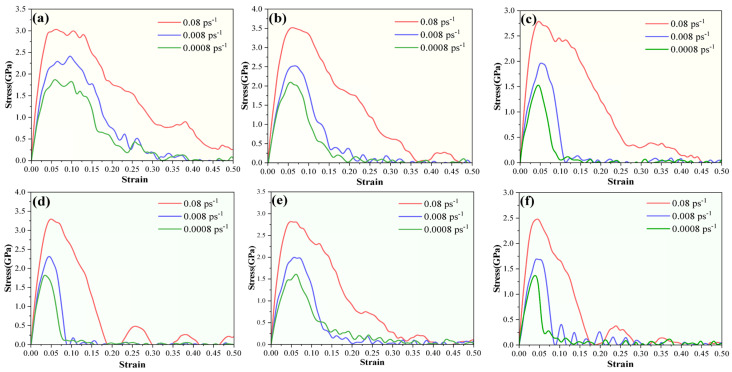
Influence of strain rate on the stress–strain relationship for interfacial composite structures, (**a**) SiO_2_-CSH_(100)-SiO_2_, (**b**) SiO_2_-CSH_(010)-SiO_2_, (**c**) SiO_2_-CSH_(001)-SiO_2_, (**d**) SiO_2_-Ca(OH)_2__(100)-SiO_2_, (**e**) SiO_2_-Ca(OH)_2__(010)-SiO_2_, and (**f**) SiO_2_-Ca(OH)_2__(001)-SiO_2_.

To investigate the influence of the strain rate on the nanomechanical behavior of the composite models with different configurations, the stress–strain curves of SiO_2_-CSH-SiO_2_ and SiO_2_-Ca(OH)_2_-SiO_2_ at strain rates of 0.08 ps^−1^, 0.008 ps^−1^ and 0.0008 ps^−1^, respectively, are compared in Figure 7. For the given interfacial composite model, the ultimate strength increased with the increasing strain rate, while the decline in residual strength was also prolonged. It can be also found that, for both hydration products, the different strain rates did not change the magnitude relationship of the ultimate stresses among the different configurations of the interfacial composite structures; i.e., the ultimate stresses of the composite structures invariably followed the relationship of *σ*_u_(010)_ > *σ*_u_(100)_ > *σ*_u_(001)_ at different strain rates. As for SiO_2_-Ca(OH)_2_-SiO_2_, different strain rates did not change the ultimate stress relationship of *σ*_u_(100)_ > *σ*_u_(010)_ > *σ*_u_(001)_. In addition, CSH tended to exhibit higher bonding properties than Ca(OH)_2_ for the interfacial composite structure, which could be reflected by the fact that, in most cases, both the ultimate strength and the integral of the stress–strain curve tended to be larger for CSH than for Ca(OH)_2_ with a particular contacted crystallographic plane and strain rate. More information related to the comparison of the nanomechanical behaviors of CSH and Ca(OH)_2_ in the interfacial composite structure are presented in Section 4.

**Figure 8:**
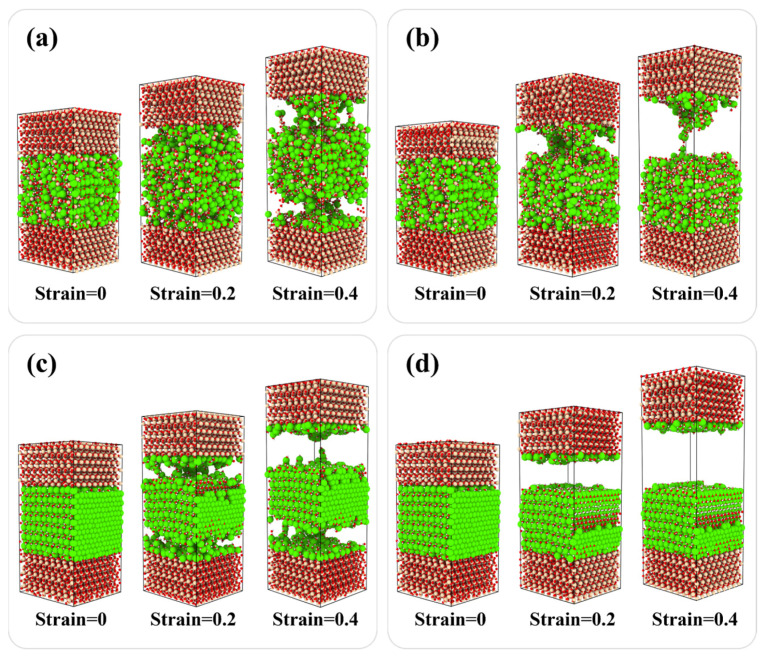
Fracture evolution for SiO_2_-CSH_(100)-SiO_2_ and SiO_2_-Ca(OH)_2__(100)-SiO_2_ at different strain rates, (**a**) SiO_2_-CSH_(100)-SiO_2_ at 0.08 ps^−1^, (**b**) SiO_2_-CSH_(100)-SiO_2_ at 0.0008 ps^−1^, (**c**) SiO_2_-Ca(OH)_2__(100)-SiO_2_ at 0.08 ps^−1^, and (**d**) SiO_2_-Ca(OH)_2__(100)-SiO_2_ at 0.0008 ps^−1^. (red spheres represent the O atoms; yellow spheres represent the Si atoms; white spheres represent the H atoms; green spheres represent the Ca atoms).

The fracture evolution for SiO_2_-CSH_(100)-SiO_2_ and SiO_2_-Ca(OH)_2__(100)-SiO_2_ at strain rates of 0.08 ps^−1^ and 0.0008 ps^−1^ are compared in Figure 8. For both CSH and Ca(OH)_2_, a high strain rate of 0.08 ps^−1^ (Figure 8a,c) resulted in simultaneous fractures at both interfaces of the composite structures. Meanwhile, compared with the strain rate of 0.0008 ps^−1^ (Figure 8b,d), more atomic chains were formed to provide a bridging effect in the composite structure during the tensile process at a strain rate of 0.08 ps^−1^, which corresponds to a higher strain rate, contributing to a more prolonged decline in the residual strength. According to previous studies, the influence of the strain rate on the fracture evaluation has been attributed as follows: as strain is transferred from the high-density region to the low-density region, the movement of the atoms results in the clustering of defects in the molecular structure, and relatively high strain rates contribute more to the development of clustering [18]. In this paper, both interfacial zones of the interfacial composite structure exhibit relatively low and approaching densities; thus, except for SiO_2_-CSH_(001)-SiO_2_, which fractured at the water layers in the CSH bulk, defects tended to develop in both interfacial zones at a relatively high strain rate.

## 4. Discussion

According to the results in this paper, it can be found that interfacial composite structures with different configurations, considering the anisotropy of CSH and Ca(OH)_2_, exhibited diverse nanomechanical behaviors in terms of their ultimate strength, stress–strain relationship and fracture evaluation. Furthermore, the strain rate also significantly affected the nanomechanical behaviors of the interfacial composite structures.

**Figure 9:**
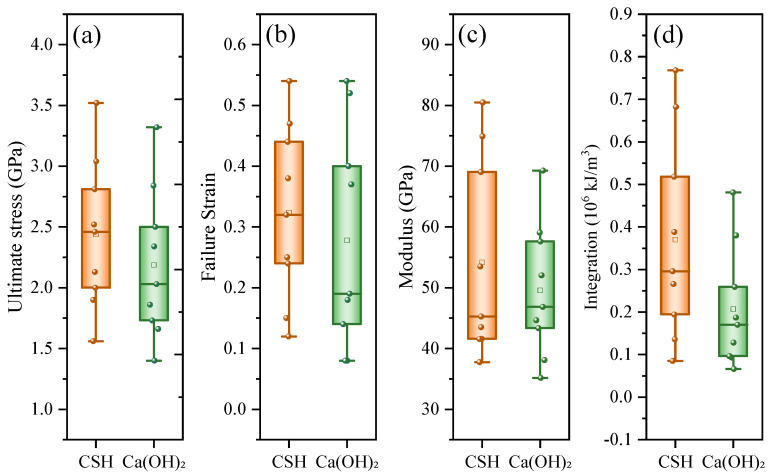
Statistical box chart for multiple nanomechanical parameters of SiO_2_-CSH-SiO_2_ and SiO_2_-Ca(OH)_2_-SiO_2_ considering the influence of configurations and strain rates. (**a**) Ultimate stress, (**b**) Failure strain, (**c**) Modulus, and (**d**) Stress–strain curve integration.

For a comprehensive comparison of the nanomechanical behaviors of CSH and Ca(OH)_2_ in interfacial composite structures, multiple nanomechanical parameters, including the ultimate strength, failure strain, modulus and integration of the stress–strain curve, of SiO_2_-CSH-SiO_2_ and SiO_2_-Ca(OH)_2_-SiO_2_ are listed in Table A1, and the results of the statistical box chart for the data in Table A1 are shown in Figure 9. In general, there were substantial regions of overlap among the box charts, and the median and mean modulus values of both interfacial composite structures appeared to be approached (refer to Figure 9c). This suggests that the ITZs composed of various hydration products in mortar/cemented soil exhibit a favorable synergistic deformation relationship. On the other hand, it is also noteworthy that, except for the median value of the modulus, the mean and median values of all nanomechanical parameters exhibited higher values for SiO_2_-CSH-SiO_2_ in comparison to those of SiO_2_-Ca(OH)_2_-SiO_2_.

Prior investigations have scrutinized the correlation between nanomechanical behavior derived from MD simulations and macroscopic performance in order to validate the fidelity of simulations [40,41]. In this paper, the calculation results can be further supported by experimental phenomena documented in the investigation of cemented soils. To be specific, several studies have investigated the incorporation of additives like fly ash to enhance the strength of cemented soils through volcanic ash reactions, which involve the consumption of Ca(OH)_2_ and the formation of additional CSH [39,42].

**Figure 10:**
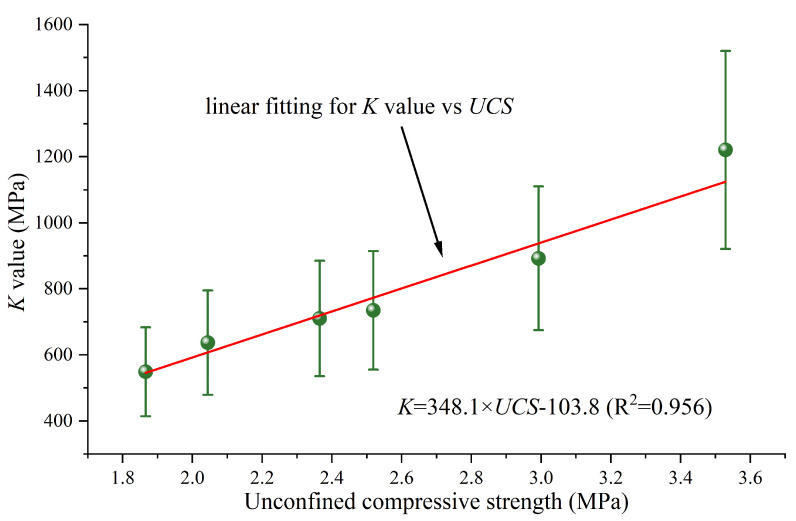
Linear relationship between the *K* value and UCS. The UCS values were obtained from the results of UCS tests as reported in Ref. [39], and the mass percentage of hydration products was determined based on the thermogravimetric curves mentioned in Ref. [39]. Meanwhile, the *K* value calculation process incorporated the standard deviation of the ultimate stress of the interfacial composite structures.

To establish the connection between the nanomechanical behavior parameters of the interfacial composite structure and the macroscopic mechanical property parameters, a nanomechanical parameter *K* was calculated according to Equation (4) and correlated with the unconfined compressive strength (UCS) of cemented soils. In Equation (4), *K* represents the nanomechanical index of the interfacial composite structure. *ω*_CSH_ denotes the mass proportion of CSH in cemented soils, while *f*_CSH_ corresponds to the average value of the ultimate stress of the interfacial composite structure SiO_2_-CSH-SiO_2_ calculated in this paper. Similarly, *ω*_Ca(OH)2_ indicates the mass proportion of Ca(OH)_2_, and *f*_Ca(OH)2_ relates to the average value of the ultimate stress of the interfacial composite structure SiO_2_-Ca(OH)_2_-SiO_2_. The correlation between *K* and UCS is illustrated in Figure 10, in which the UCS values were obtained from the results of UCS tests as reported in Ref. [39], and the mass percentage of hydration products was determined based on the thermogravimetric curves mentioned in Ref. [39]. By examining the correlation in Figure 10, it can be found that there was a linear increasing relationship between the *K* values and UCS. The superior linear fitting results suggest a robust correlation between the *K* value, accounting for the composition of hydration products and the nanomechanical behavior of the interfacial composite structure, and the UCS. Additionally, it is also shown that the improved interfacial interaction at the nanoscale of the cemented soils results in an increase in macroscopic strength. While certain studies ascribe this strength improvement to a reduction in porosity, others have reported significant strength increases despite a limited reduction in porosity associated with a higher CSH content [43].

*K* = *ω*_CSH_ × *f*_CSH_ + *ω*_Ca(OH)_2__ × *f*_Ca(OH)_2__ (4)

**Figure 11:**
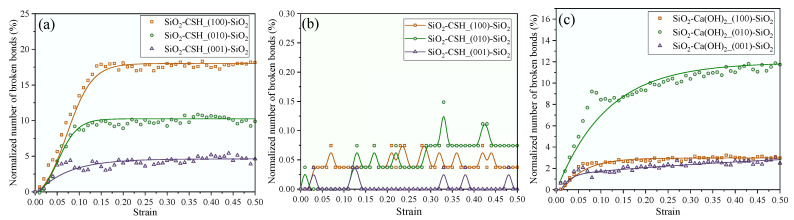
Normalized number of broken bonds for interfacial composite structures at 0.008 ps^−1^, (**a**) Ca-O bonds in SiO_2_-CSH-SiO_2_, (**b**) Si-O bonds in SiO_2_-CSH-SiO_2_, and (**c**) Ca-O bonds in SiO_2_-Ca(OH)_2_-SiO_2_.

**Figure A1:**
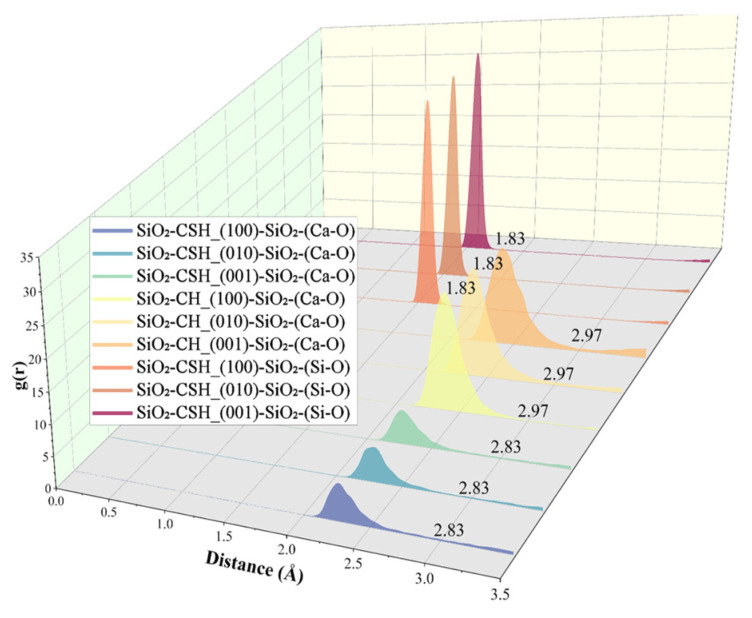
RDF curves of interfacial composite structures.

To further understand the fracturing process of the interfacial composite structure, the criterion for broken atom pairs was determined according to the RDF calculation results of Ca-O and Si-O in the interfacial composite structures (see Figure A1), and the normalized number of broken bonds of the different configurations are compared in Figure 11. As can be seen from Figure 11a, the process of the tensile fracturing of SiO_2_-CSH-SiO_2_ structures with different configurations is associated with visible Ca-O bond breakage. Through a combined examination of the stress–strain curves depicted in Figure 4 and the integration values provided in Table A1, a notable relationship is observed: a higher normalized number of Ca-O after the fracturing of the composite structure corresponded to a slower decay rate of the residual strength and a higher integration value within the stress–strain curve. In contrast, normalized numbers of the Si-O bond in different configurations remained at low levels as shown in Figure 11b, which implies that there was almost no fracturing of the Si-O bond in the SiO_2_-CSH-SiO_2_ structure during the tensile process. In Figure 11c, the normalized number of broken Ca-O bonds is presented for the SiO_2_-Ca(OH)_2_-SiO_2_ structure. Remarkably, similar to the behavior observed in the SiO_2_-CSH-SiO_2_ structure, the number of broken Ca-O bonds within the SiO_2_-Ca(OH)_2_-SiO_2_ structure with various configurations exhibited a gradual increase during the tensile process. It is important to highlight that the SiO_2_-Ca(OH)_2__(010)-SiO_2_ configuration, which had the highest normalized number of Ca-O bonds broken after the complete fracturing of the interfacial composite structure, also displayed the slowest decline in residual strength and the highest value for the integral area of the stress–strain curve. In contrast, the other two configurations of SiO_2_-Ca(OH)_2_-SiO_2_ demonstrated a relatively rapid decrease in residual strength and had smaller integral areas in the stress–strain curve. According to previous studies, the decay of the residual strength of the molecular model can characterize the plastic damage at the nanoscale [44], while the integral area of the stress–strain curve corresponds to the energy consumption during the damage process [45]. The comprehensive fracture normalized number comparison results show that for both SiO_2_-CSH-SiO_2_ and SiO_2_-Ca(OH)_2_-SiO_2_, the tensile process of the interface composite structure mainly involves the breakage of the Ca-O bond, and the number of broken Ca-O bonds determines the plastic damage characteristics of the model at the nanoscale and the energy consumption in the tensile process.

**Figure 12:**
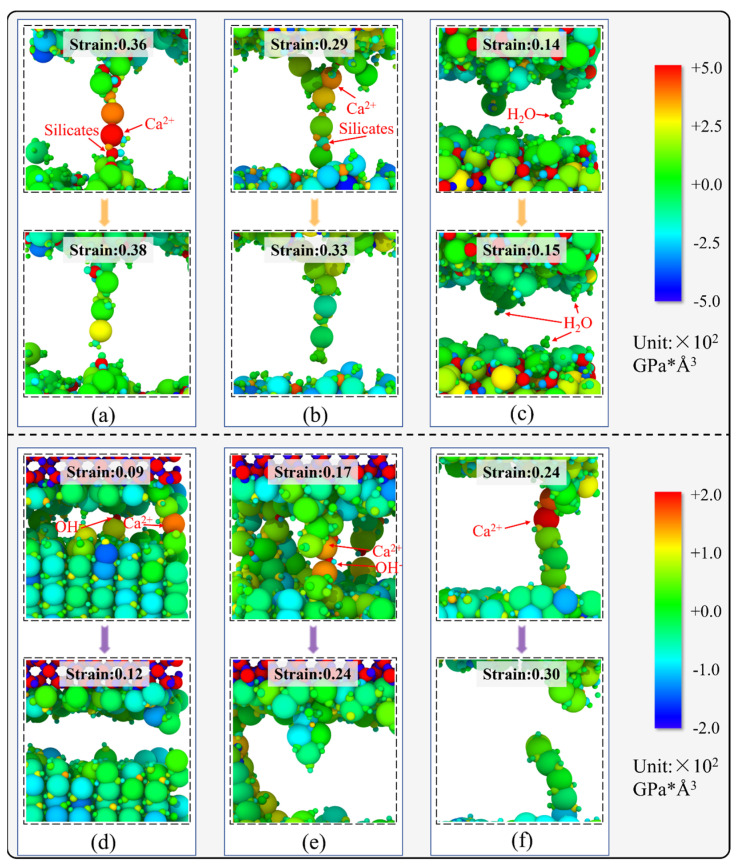
Per-atom stress map at the local scale during tensile loading, (**a**) SiO_2_-CSH_(100)-SiO_2_, (**b**) SiO_2_-CSH_(010)-SiO_2_, (**c**) SiO_2_-CSH_(001)-SiO_2_, (**d**) SiO_2_-Ca(OH)_2__(100)-SiO_2_, (**e**) SiO_2_-Ca(OH)_2__(010)-SiO_2_, and (**f**) SiO_2_-Ca(OH)_2__(001)-SiO_2_.

Understanding atomic chain bridging is crucial for insights into the decline in strength in interfacial composite structures. Moreover, analyzing the fracture characteristics of atomic chains can also verify the results obtained from calculations of the normalized number of broken bonds at the local scale. Figure 12 depicts the per-atom stress mapping of the local structures during the tensile loading of SiO_2_-CSH-SiO_2_ and SiO_2_-Ca(OH)_2_-SiO_2_ with different configurations at a strain rate of 0.008 ps^−1^. As shown in Figure 12a,b, atomic chains of SiO_2_-CSH_(100)-SiO_2_ and SiO_2_-CSH_(010)-SiO_2_ were composed of Ca^2+^ ions and silicates. Meanwhile, both Ca^2+^ ions and silicates experienced stress concentrations when the atomic chain was stretched, and the stress of Ca^2+^ ions was significantly released when the atomic chain was broken. According to Figure 12c, it can be seen that the bridging atomic chain of SiO_2_-CSH_(001)-SiO_2_ mainly consisted of water molecules which were significantly weaker in elongation than that of the atomic chain composed of Ca^2+^ ions and silicates. It can also be noted that the fracture surface of SiO_2_-CSH_(001)-SiO_2_ was mainly composed of exposed water molecules, which is consistent with the characterization of the fracture damage of CSH at the internal water layer in previous studies [18,43]. On the other hand, in the SiO_2_-Ca(OH)_2_-SiO_2_ composite structure (see Figure 12d–f), the atomic chain of all the configurations consists of Ca^2+^ ions and hydroxyl groups (OH^−^). Furthermore, Ca^2+^ ions tended to be the atoms with concentrated stress during the tensile process in the SiO_2_-Ca(OH)_2_-SiO_2_ composite structure. Combining local-scale stress in the interfacial composite structures of CSH and Ca(OH)_2_ with different configurations, it is found that the rupture of the Ca-O bond as the primary atomic pair during the tensile process can also be reflected by the broken characteristics of the atomic chain in the per-atom stress map.

## 5. Conclusions

Regarding the interfacial composite structure between the main hydration products of OPC (CSH and Ca(OH)_2_) and SiO_2_, tensile tests were performed using MD simulations to investigate the influence of the anisotropy of the hydration products on the nanomechanical behavior at different strain rates. Our conclusions were drawn as follows:

1. In interfacial composite structures of SiO_2_-CSH-SiO_2_ and SiO_2_-Ca(OH)_2_-SiO_2_, the interfacial zones (IZs) tended to have relatively lower densities than those of the bulk. Meanwhile, the anisotropy of the hydration products had almost no effect on the IZ being a low-density zone. For SiO_2_-CSH-SiO_2_, areas with elevated water densities in the bulk CSH also corresponded to lower overall densities.
2. Interfacial composite structures with different configurations, considering the anisotropy of CSH and Ca(OH)_2_, exhibited diverse nanomechanical behaviors in aspects of their ultimate strength, stress–strain relationship and fracture evaluation. The SiO_2_-CSH_(100)-SiO_2_ configuration exhibited fracturing within the water layer situated within the bulk of CSH, leading to diffusion rates of H_2_O molecules and Ca^2+^ ions in the interfacial zone (IZ) that were consistent with those observed in the bulk. In contrast, other interfacial composite structures fractured in proximity to the IZ, resulting in the significantly accelerated diffusion of H_2_O molecules/OH^−^ ions and Ca^2+^ ions within the IZ compared to the bulk region.
3. For all configurations of the interfacial composite structures, a higher strain rate contributed to a higher ultimate strength and a more prolonged decline in the residual strength. In terms of the evolution of fractures, a relatively high strain rate (0.08 ps^−1^) induced concurrent fractures at both interfaces of the composite structures and also resulted in more atomic chains to provide a bridging effect as opposed to a lower strain rate (0.0008 ps^−1^).
4. In the interfacial composite structures, both CSH and Ca(OH)_2_ exhibited the rupturing of the Ca-O bond as the primary atomic pair during the tensile process, which can also be reflected by the broken characteristics of the atomic chain in the per-atom stress map. The plastic damage characteristics of the interfacial composite structures and the energy consumption during the tensile process can be effectively assessed by analyzing the normalized number of broken Ca-O bonds.

## Data Availability

The data supporting the findings of this study are available by reasonable request to zhaojiuye@dlmu.edu.cn.

## Appendix A

**Table A1:** Multiple parameters for the nanomechanical behavior of SiO_2_-CSH-SiO_2_ and SiO_2_-Ca(OH)_2_-SiO_2_.

Hydration Product	Strain Rate	Plane	Ultimate Stress (GPa)	Failure Strain	Modulus (GPa)	Integration (10^6^ kJ/m^3^)
CSH	0.08	(100)	3.04	0.54	74.92	0.768
		(010)	3.52	0.47	80.49	0.682
		(001)	2.81	0.44	69.04	0.518
	0.008	(100)	2.46	0.38	45.26	0.388
		(010)	2.52	0.25	53.5	0.266
		(001)	2.00	0.12	41.58	0.136
	0.0008	(100)	1.90	0.32	41.55	0.296
		(010)	2.13	0.24	43.5	0.194
		(001)	1.56	0.15	37.76	0.085
Ca(OH)_2_	0.08	(100)	3.32	0.52	69.27	0.380
		(010)	2.84	0.54	59.07	0.481
		(001)	2.50	0.40	52.03	0.259
	0.008	(100)	2.34	0.08	57.60	0.128
		(010)	2.03	0.19	46.84	0.187
		(001)	1.73	0.08	43.34	0.093
	0.0008	(100)	1.86	0.14	44.65	0.096
		(010)	1.66	0.37	38.10	0.170
		(001)	1.40	0.18	35.16	0.066
